# A systematic mutational analysis identifies a 5‐residue proline tag that enhances the *in vivo* immunogenicity of a non‐immunogenic model protein

**DOI:** 10.1002/2211-5463.12941

**Published:** 2020-08-30

**Authors:** Nafsoon Rahman, Mohammad Monirul Islam, Md. Golam Kibria, Satoru Unzai, Yutaka Kuroda

**Affiliations:** ^1^ Department of Biotechnology and Life Sciences Graduate School of Engineering Tokyo University of Agriculture and Technology Tokyo Japan; ^2^ Department of Biochemistry and Molecular Biology University of Chittagong Chittagong Bangladesh; ^3^ Department of Frontier Bioscience Faculty of Bioscience and Applied Chemistry Hosei University Tokyo Japan

**Keywords:** immunogenicity, monomer, peptide tag, protein solubility, SCP

## Abstract

Poor immunogenicity of small proteins is a major hurdle in developing vaccines or producing antibodies for biopharmaceutical usage. Here, we systematically analyzed the effects of 10 solubility controlling peptide tags (SCP‐tags) on the immunogenicity of a non‐immunogenic model protein, bovine pancreatic trypsin inhibitor (BPTI‐19A; 6 kDa). CD, fluorescence, DLS, SLS, and AUC measurements indicated that the SCP‐tags did not change the secondary structure content nor the tertiary structures of the protein nor its monomeric state. ELISA results indicated that the 5‐proline (C5P) and 5‐arginine (C5R) tags unexpectedly increased the IgG level of BPTI‐19A by 240‐ and 73‐fold, respectively, suggesting that non‐oligomerizing SCP‐tags may provide a novel method for increasing the immunogenicity of a protein in a highly specific manner.

## Graphical abstract

Solubility controlling peptide tags (SCP‐tags) constructed with 5‐proline (C5P) and 5‐arginine (C5R) residues did not alter the secondary structure content nor the tertiary structure nor the monomeric state of BPTI‐19A, but significantly increased its *in vivo* immunogenicity, by up to 240‐ and 73‐fold, respectively, suggesting the potential of non‐oligomerizing SCP‐tags for increasing the immunogenicity of a non‐immunogenic protein.

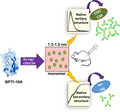

**Abbreviations**
AUC: analytical ultra‐centrifugation
BPTI‐19A (untagged BPTI‐19A): a simplified variant of bovine pancreatic trypsin inhibitor containing 19 alanines
BPTI‐C5P: a BPTI‐19A with C5P tag at its C terminus; other tags are named correspondingly
DLS: dynamic light scattering
SCP‐tags: solubility controlling peptide tags
SLS: static light scattering

The poor immunogenicity of small low molecular weight proteins [1] represents a significant challenge in producing antibodies for biopharmaceutical usage as well as for developing protein‐based vaccines. Adjuvants are commonly used for addressing this issue, but in biomedical practice, they often encounter a patients' reluctance to use such additives [2, 3]. Besides adjuvants, virus‐like particles (VLPs) have recently earned much attention in increasing the immunogenicity of proteins and a malaria vaccine candidate is being reported [4]. The fusion protein strategy, where the target protein is fused to a different protein, is another popular way for increasing immunogenicity. For example, hydrophobic lipoproteins [5], cytokines [6], or immunoglobulin domains [7] have been used to enhance the immunogenicity of target proteins. Other methods such as nanoparticles [8], carrier proteins [9], and bioconjugates [10] are also known for increasing a protein's immunogenicity. Nevertheless, none of the above methods is perfect, and the biopharmaceutical industry is continuously in search of a novel, simple, and widely applicable method for increasing the immunogenicity of proteins.

In previous reports, we showed that short solubility controlling peptide tags (SCP‐tags) [11, 12, 13] attached at the C terminus of a host protein could be used to induce the formation of subvisible amorphous aggregates and thereby increase the immunogenicity of non‐immunogenic proteins [14, 15]. In particular, a hydrophobic isoleucine tag attached to the C terminus of two model proteins, BPTI‐19A (58 residues; MW: 5.98 kDa) and DEN3ED3 (103 residues; MW 11.46 kDa), increased their immunogenicity by oligomerizing the proteins into small nanometer‐scale aggregates, and the immune response was long‐lasting with a T‐cell‐dependent activation of the B cells [16, 17]. A major advantage of the SCP‐tags is their ability to produce subvisible aggregates in a highly reproducible and reliable way [18, 19], which in turn provides good control of their effect on immunogenicity [15].

Here, we ask whether short peptide tags that increase or did not affect a protein's solubility and thus prevent protein oligomerization can nevertheless increase a protein's immunogenicity [20, 21]. We thus systematically analyzed the effect of the following 10 SCP‐tags: Six tags made of five consecutive Arg, Lys, His, Asp, Asn, Pro; one made of seven Pro; two tags made of consecutive Arg‐lle and Asn‐Ile, all attached at the C terminus of BPTI‐19A; and a 5‐proline tag attached at the N terminus. To this end, we confirmed that all of the BPTI variants were monomeric and retained a native conformationand biophysical properties. We then measured their immunogenicity in mice and found that C5P and C5R tags significantly enhanced the anti‐BPTI‐19A IgG titer, which lasted for several weeks. Thus, in addition to the previously reported C5I tag, which increased immunogenicity through protein oligomerization, C5P and C5R tags are expected to provide a tool for increasing a protein's immunogenicity without any oligomerization. [14].

## Materials and methods

### Protein expression and purification

**Fig. 1:**
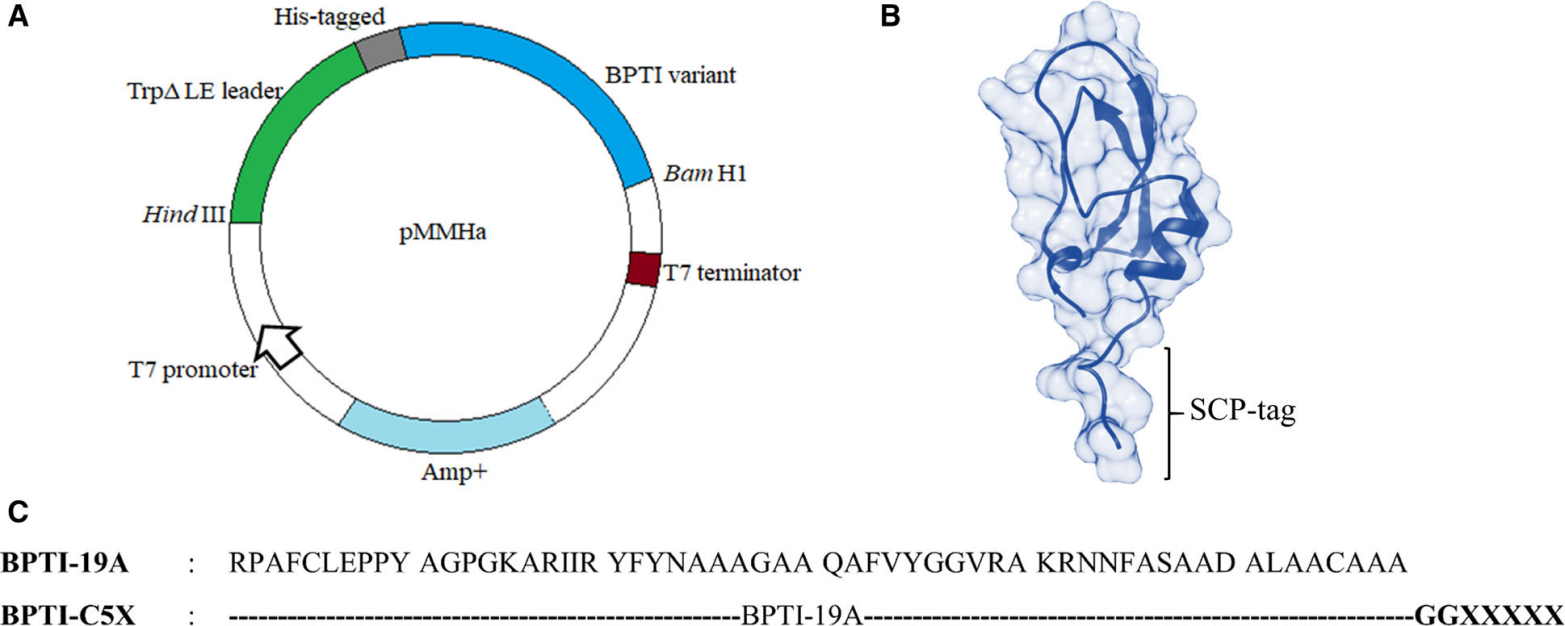
Construction and design of the tagged BPTI. (A) BPTI was expressed using the pMMHa vector with a His‐Trp leader. (B) The tagged variants were designed by attaching the respective tags at the C terminus, where two glycine residues served as a spacer between the target protein and the tags. The structure of SCP‐tagged BPTI was generated using MODELLER using the BPTI‐19A structure as a template (PDB ID: 3AUB) [43]. (C) The sequence of BPTI‐19A and the tagged variants. X stands for the one‐letter amino acid code and is either R, K, N, H, D, P or I.

The SCP‐tagged BPTI variants were constructed by introducing the SCP‐tags to either the N or C termini of BPTI‐19A, a simplified BPTI variant [22] containing 19 alanines out of its 58 residues [23] (Fig. 1). BPTI variants were named according to the number and type of amino acids added to the N or C terminus of BPTI‐19A. The DNA sequences encoding the SCP‐tags were added by QuikChange site‐directed mutagenesis (Stratagene, California, USA) using pMMHa BPTI‐19A vector as a template [23], and the plasmid sequences were confirmed by DNA sequencing. The tagged variants were expressed in *Escherichia coli* BL21 (DE3) pLysS cell lines as inclusion bodies and were solubilized in 6 M GuHCl with overnight oxidation at 25 °C. CNBr (cyanogen bromide) reaction was then performed in order to cleave the TrpΔLE leader sequence, and proteins were purified by pI precipitation followed by reverse phase HPLC [18]. Furthermore, protein identities were confirmed by MALDI‐TOF mass spectrometry (AB SCIEX TOF/TOF TM 5800, Framingham, USA), and the purified proteins were preserved at −30 °C as lyophilized powders. A schematic representation of protein purification is given in Fig. S4.

### Endotoxin assay

Limulus amebocyte lysate (LAL) endotoxin assay was performed following the standard protocol provided by GenScript ToxinSensor™ Chromogenic LAL Endotoxin Assay Kit (New Jersey, USA). The endotoxin levels for the tagged variants were found in the ranges of 1.36–4.73 EU·kg^−1^·h^−1^ that was below the United States Pharmacopeia <85> chapter's limit for injectable solutions of 5 EU·kg^−1^·h^−1^ [24] (Table S3).

### Dynamic light scattering and static light scattering

The hydrodynamic radii of the BPTI‐19A variants were measured at 25 °C and 37 °C by dynamic light scattering (DLS) on a Zetasizer Nano‐S (Malvern, UK) [25]. The protein samples were prepared in PBS, pH 7.4 (filtered using 0.22‐µm Minisart filter) at a concentration of 0.3 mg·mL^−1^, left still for 20 min at 25 °C, and centrifuged (20 000 ***g*** for 20 min at 25 °C). The supernatant was used for DLS and subsequent measurements as well as for immunization. Three independent readings were recorded for measuring the average hydrodynamic radius (*R*_h_) from the number distributions using the Stokes–Einstein equation [26].

The presence of subvisible aggregates was monitored by static light scattering (SLS) at a wavelength of 600 nm using an FP‐8500 spectrofluorometer (JASCO, Tokyo, Japan). A quartz cuvette with a 3‐mm optical path length was used. Each measurement was repeated three times, and the values were averaged.

### Analytical ultra‐centrifugation

Sedimentation velocity experiments were carried out using an Optima XL‐A analytical ultracentrifuge (Beckman‐Coulter, California, USA) with a four‐hole An60Ti rotor at 33 °C. Analytical ultra‐centrifugation (AUC) samples were prepared in the same way as the DLS/inoculation samples followed by overnight dialysis against PBS. PBS density, viscosity, and BPTIs' partial specific volumes were calculated using the SEDNTREP software [27]. Data analysis was performed using the continuous distribution *c* (s) analysis module in the SEDFIT program [28]. As a positive control, we used BPTI‐C5I, which was previously reported to produce subvisible oligomers [15, 18].

### Tyrosine fluorescence and CD spectroscopy

The secondary structure contents were characterized by CD spectroscopy using a JASCO J820 CD spectropolarimeter (JASCO), and spectra were measured in the wavelength range of 200–260 nm using a 1‐mm optical path length quartz cuvette. Tyr‐fluorescence spectra with λ_ex_ 275 nm were measured on a JASCO FP‐8500 spectrofluorometer using a quartz cuvette with a 3‐mm optical path length. Both measurements were conducted at a protein concentration of 0.3 mg·mL^−1^ in PBS, pH 7.4 at 25 °C and 37 °C.

### 8‐Anilino‐1‐naphthalene‐sulfonic acid fluorescence and thioflavin T assay

The native‐like conformations of the BPTI variants were further assessed by 8‐anilino‐1‐naphthalene‐sulfonic acid (ANS) and thioflavin T (ThT) fluorescence measurements under conditions identical to that of the immunization experiments. Both measurements were carried out using the JASCO FP‐8500 spectrofluorometer (JASCO) with a quartz cuvette of 3‐mm optical path length at 25 °C and 37 °C. ANS/ThT dye was added to the protein solution and incubated at 25 °C for 5 min in the dark before measurement. The ANS and ThT fluorescence spectra were measured with an excitation wavelength (λ_ex_) set to, respectively, 380 nm [29], and 440 nm [30]. As a positive control for amyloid formation, the hen egg‐white lysozyme was used in the ThT measurement [31, 32].

### Immunization experiments

Four‐week‐old female mice (outbred Jcl:ICR, CLEA, Tokyo, Japan) were used for the immunization experiments. For injections with adjuvant, the first dose was given subcutaneously with Freund's complete adjuvant (WAKO, Saitama, Japan), and doses 2–4 with Freund's incomplete adjuvant were provided intraperitoneally at weekly intervals. The quantity of injected protein was 30 µg and formulated in 100 µL of PBS or mixed with a 1 : 1 ratio of adjuvant when the latter was used. Injections without adjuvant were administrated subcutaneously with 30 µg of protein formulated in 100 µL of PBS, four times on a weekly base. Control mice were injected with PBS both in the presence and in the absence of adjuvant. Three days after inoculation, ~ 20 µL of tail‐bleed samples was collected and used for the weekly measurement of IgGs by ELISA. After the final dose, the mice were sacrificed, and heart‐bleed samples were collected and stored at −30 °C with 1 : 4 dilution in PBS supplemented with 20% glycerol. All of the animal experiments were performed in compliance with the Tokyo University of Agriculture and Technology (TUAT) review panel for animal experimentation and the Japanese governmental regulations.

### ELISA

ELISA was performed as previously reported [14, 33]. In short, anti‐BPTI IgG levels were evaluated using untagged BPTI‐19A (2.5 µg·mL^−1^ in PBS) as coating antigen on the 96‐well microtiter plates (TPP microtiter plates, Japan). Anti‐BPTI sera were applied into the PBS‐washed wells at an initial dilution of 1 : 50 and 1 : 300 for the tail‐bleed and heart‐bleed samples, respectively. Plates were then incubated at 37 °C for 2 h.

After washing the plates three times with PBS‐0.05% Tween‐20, each well received a 100 μL of anti‐mouse IgG HRP conjugate (Thermo Fisher Scientific, Waltham, USA) at 1 : 3000 dilution in 0.1% BSA‐PBS‐Tween‐20 and incubated at 37 °C for 1.5 h. As a substrate, *O*‐phenyl Di‐amine (OPD) was added. The color intensities were measured at 492 nm using a microplate reader (SH‐9000 Lab, Hitachi High‐Tech Science, Tokyo, Japan) immediately after stopping the reaction with 1 N sulfuric acid (50 µL/well).

## Results and Discussion

### Biophysical characterization of the tagged BPTIs

The five‐residue SCP‐tags used in this study are found to increase or barely changed the solubility of our model protein, the untagged BPTI‐19A, when attached to its C terminus [20, 21]. In brief, the Arg, Lys, His (positively charged), and Asn (neutral) tags enhance the solubility of BPTI regardless of the pH, whereas an Asp tag increases the solubility in a pH‐dependent manner (due to the ionization of the side chain). To note, the solubility of BPTI was unaffected by the Pro tag. Finally, we designed an N‐terminal 5‐pro tag and a C‐terminal 7‐pro tag to examine the effects of the proline tag's position and length on the immune responses of BPTI‐19A. All samples were prepared in PBS and used under the same conditions as for the biophysical characterization (see details of the preparation in the Materials and methods).

**Fig. 2:**
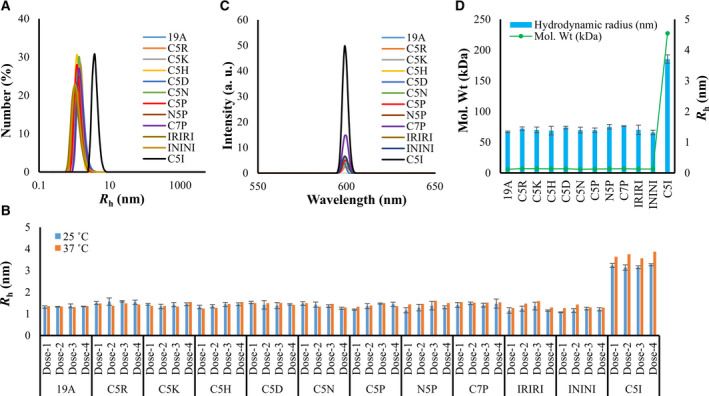
Analysis of the monomeric and oligomeric status of the tagged and untagged‐19A under physiological conditions. (A) Number distribution of the hydrodynamic radii (*R*_h_) measured by DLS. (B) *R*_h_ measured just before immunization from dose‐1 to dose‐4 and computed from DLS's number distribution. (C) SLS spectra of the BPTI variants. (D) Molecular weights of tagged variants measured at 33 °C by AUC and their correlation with the *R*_h_. DLS/SLS data were the averages of three independent measurements. Line symbols are explained within the panels. The error bars represent the standard deviation.

The sizes of the tagged BPTI variants were systematically examined by DLS and SLS. DLS results showed that all tagged BPTIs were monomeric at both temperatures (Fig. 2A, Table S1 and Fig. S1A), which was confirmed by the low scattering intensities as measured by SLS (Fig. 2C and Fig. S1B). AUC results also confirmed the monomeric status of all of the tagged BPTIs as they showed single peaks with an average molecular weight of ~ 6.6 kDa (very close to that of the untagged BPTI‐19A; 6.1 kDa) (Table 1 and Fig. S5). In particular, BPTI‐C5P and BPTI‐C5R had a monomeric population of 98.1% and 97.2%, respectively, and all other SCP‐tagged variants had > 90% of monomeric population (Table 1). In this study, we used AUC for characterizing the aggregation states of the mutants, because it is considered as a powerful and accurate method that reliably detects aggregates without the need of interacting matrices. Indeed, size exclusion chromatography (SEC) often fails to detect the actual aggregate contents due to their interaction with the SEC‐column matrix [34, 35].

**Table 1:** Molecular weights of BPTI‐19A variants by AUC/Sedimentation velocity. AUC experiments were carried out with a protein concentration of 0.3 mg·mL^−1^ in PBS at 33 °C. The *c* (s) distribution was converted into a molar mass distribution *c* (M), from which the average molecular weights were calculated.

Mutant identities	Molecular weights (kDa)	Monomeric population (%)
BPTI‐19A	6.1	90.1
BPTI‐C5R	7.0	98.1
BPTI‐C5K	6.9	96.2
BPTI‐C5H	6.7	95.2
BPTI‐C5D	6.8	97.2
BPTI‐C5N	6.1	74.7
BPTI‐C5P	6.4	97.2
BPTI‐N5P	6.6	91.7
BPTI‐C7P	6.9	90.7
BPTI‐IRIRI	6.6	95.0
BPTI‐ININI	6.5	94.7
BPTI‐C5I	Large^a^	a

^a^ The molecular weight of BPTI‐C5I could not be determined as it formed oligomers with multiple sizes.

Altogether, all of the DLS, SLS, and AUC data indicated that the SCP‐tagged BPTIs remained monodispersed and monomeric in solution alike the untagged BPTI‐19A (Fig. 2A–D). As control of an aggregated mutant, we used the C5I‐tagged BPTI, which formed oligomers with a distribution of sizes (Fig. 2A–D), in line with our previous report [15, 18]. In addition to the AUC results, which unambiguously showed that the proteins were in a monomeric state, DLS was repeatedly measured before each round of injection (i.e., a “near‐real‐time” monitoring' of *R*_h_) in order to fully ensure that the tagged samples (except BPTI‐C5I) were actually monomeric at the time of injection (Fig. 2B).

**Fig. 3:**
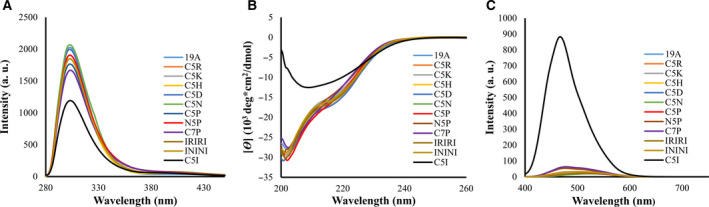
Structural characterization of the BPTI‐19As under physiological conditions. (A) Tyr fluorescence, (B) CD, and (C) ANS fluorescence spectra measured at a protein concentration of 0.3 mg·mL^−1^ in PBS, pH 7.4. Data are the averages of three accumulations. Line symbols are explained within the panels.

CD indicated that the tagged BPTI variants had a secondary structure content similar to that of the untagged BPTI‐19A, and Tyr fluorescence indicated that they retained a native‐like tertiary structure (Fig. 3A,B and Fig. S1C,D). Furthermore, ANS fluorescence intensity of the untagged‐19A and all other variants remained very low compared to that of BPTI‐C5I (Fig. 3C and Fig. S1E), confirming their native‐like structural properties. Moreover, no ThT fluorescence signal was observed indicating the absence of amyloidogenic aggregates [29, 36] (Fig. S1F). Altogether, all of the measurements indicated that none of the tags (except the previously reported C5I) affected the conformation nor the monomeric state of BPTI‐19A under injecting conditions.

### Search for a non‐aggregating immunogenicity‐enhancing peptide tag

**Fig. 4:**
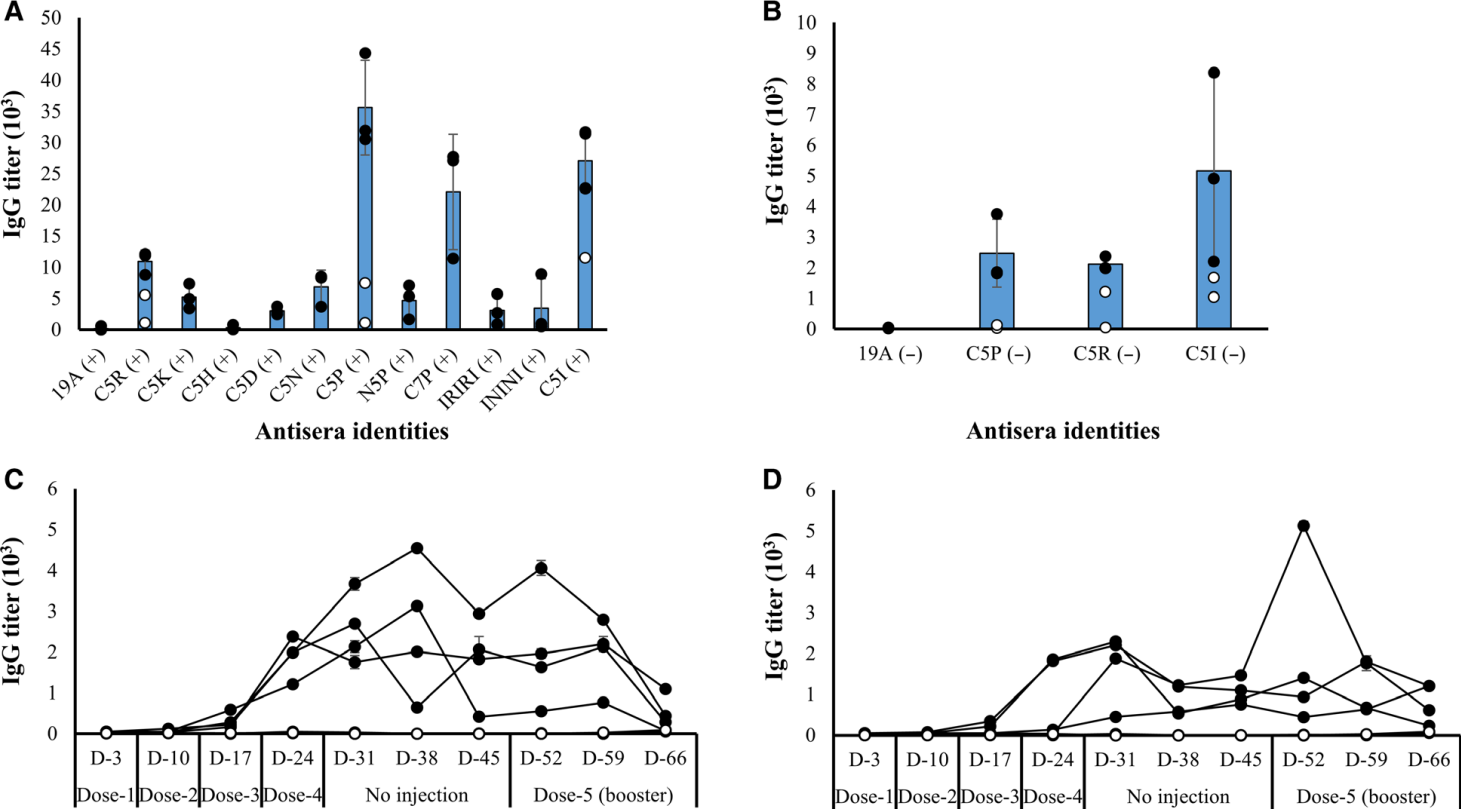
Antibody responses against the tagged and the untagged BPTI‐19A. Immunization in the presence and absence of adjuvant are indicated, respectively, by ‘+’ and ‘−’. (A) IgG titers after the final dose (4th dose) were measured using the tail‐bleed sera of mice in the presence of adjuvant. Outliers (open circles) were removed when computing the average IgG titer (gray bars), which was calculated using data from the high responsive mice (closed circles) [19A (+), *n* = 4, C5P/R/I (+), *n* = 5, C5K/D/N/H/IRIRI/ININI (+), *n* = 3, N5P/C7P (+), *n* = 3; ‘*n*’ indicates the number of mice]. (B) Antibody titers of BPTI‐19A, BPTI‐C5P, BPTI‐C5R, and BPTI‐C5I in the absence of adjuvants after the final (4th dose) using the tail‐bleed sera. The average IgG titer (gray bars) was calculated using data from high responsive mice (closed circles) and the outliers were excluded (open circle) [19A (−), *n* = 3; C5P/R/I (−), *n* = 5]. Long‐term IgG titers against (C) untagged‐19A (open circle), BPTI‐C5R (closed circles), and (D) BPTI‐C5P (closed circles) in the absence of adjuvant [19A (−), *n* = 3 and C5P/R (−), *n* = 4]. ‘D’ indicates the day at which tail bleeding was performed counting from the first inoculation. A booster dose was given on D‐45, and the IgG titer on D‐52 indicates the immune response a week after the booster dose was administrated. Line symbols are explained within the panels. The error bars represent the standard deviation.

We investigated the immunogenicity of all of the 10 tagged BPTI‐19A in Jcl:ICR mice model both in the presence and in the absence of adjuvant. The untagged BPTI‐19A, which remained monomer, was non‐immunogenic as expected for a small 6‐kDa protein (Fig. 4), even in the presence of Freund's adjuvant. However, the antibody titers of some of the tagged BPTI‐19A variants became noticeable after the third injection (Fig. S2). In particular, after the final dose (4th), the titer of the anti‐BPTI‐C5P was 240‐fold higher than that of the untagged BPTI‐19A (Fig. 4A, Fig. S3A). This increase was even higher than the 182‐fold increase observed in our control experiment, where the oligomerizing C5I tag was attached to BPTI‐19A (Fig. 4A and Table 2). To date, the C5R tag, which also kept BPTI‐19A monomeric at the time of injection, increased the immunogenicity of BPTI‐19A by 73‐fold. Interestingly, N5P barely increased BPTI‐19A's immunogenicity, and C7P increased the immune response by 149‐fold, which was a substantial increase but less than we expected from the increase resulting from the C5P tag (Fig. 4A and Table 2).

**Table 2:** Average IgG titer values for tagged BPTI variants with and without adjuvant.

Dose formulation	Mutants	Tags	Average IgG titers ^a^	Fold increased^b^
With adjuvant (+)	BPTI‐19A	×	148.3	1
	BPTI‐C5R	Gly_2_ Arg_5_	10946.9	73.8
	BPTI‐C5K	Gly_2_Lys_5_	5252.4	35.4
	BPTI‐C5H	Gly_2_His_5_	302.9	2.04
	BPTI‐C5D	Gly_2_Asp_5_	3020.9	20.4
	BPTI‐C5N	Gly_2_Asn_5_	6858.9	46.3
	BPTI‐C5P	Gly_2_Pro_5_	35587.2	240.0
	BPTI‐N5P	Pro_5_ Gly_2_	4722.5	31.9
	BPTI‐C7P	Gly_2_Pro_7_	22093.1	149.0
	BPTI‐IRIRI	Gly_2_Ile_3_Arg_2_	3115.3	21.0
	BPTI‐ININI	Gly_2_Ile_3_Asn_2_	3470.4	23.4
	BPTI‐C5I	Gly_2_Ile_5_	27110.9	182.9
Without adjuvant (−)	BPTI‐19A	×	30.6	1
	BPTI‐C5R	Gly_2_ Arg_5_	2121.3	69.4
	BPTI‐C5P	Gly_2_Pro_5_	2481.2	81.2
	BPTI‐C5I	Gly_2_Ile_5_	5163.3	168.9

^a^ The titers were calculated using a power fitting model, and the values were averaged using the number of highest responsive mice in respective groups [with adjuvant group: 19A (+), *n* = 4, C5P/R/I (+), *n* = 5, C5K/D/N/H/IRIRI/ININI (+), *n* = 3, N5P/C7P (+), *n* = 3; without adjuvant group: 19A (−), *n* = 3; C5P/R/I (−), *n* = 5; where ‘*n*’ indicates the number of mice].

^b^ The fold increase of the titers in the presence and absence of adjuvant was calculated with respect to the titer of BPTI‐19A (+) and BPTI‐19A (−), respectively. ‘×’ indicates the absence of tags.

Immunization experiments in the absence of adjuvant were carried out for tags that showed the highest immune response. Namely, the C5P and C5R‐tagged BPTI‐19As, the untagged BPTI‐19A, and the oligomerizing C5I‐tagged BPTI‐19A were used. Noteworthy and in line with our previous observation, the C5I tag increased the antibody titer of BPTI‐19A by 168‐fold, reflecting the importance of the nanometer size aggregates in increasing a protein's immunogenicity (Fig. 4B and Table 2). C5P and C5R tags significantly increased the immunogenicity of the BPTI‐19A by 81‐ and 69‐fold, respectively. Importantly, all of the above ELISA figures remained essentially unchanged whenever the plates were coated with the tagged BPTIs (own‐tags) or the untagged BPTI‐19A (Fig. S3C) indicating that the IgGs were raised against BPTI‐19A itself and not the tags. Furthermore, the effect of the tags was cumulative to that of the Freund's adjuvant, indicating the tag's strong potential for acting as a target‐specific adjuvant (Fig. S3B).

Finally, we assessed the maintenance of the immune responses generated by the untagged‐19A, BPTI‐C5P, and BPTI‐C5R and monitored the IgG level for several weeks after the last injection. Mice were weekly injected four times with the corresponding variant in the absence of adjuvant and kept untreated for 3 weeks, during which the IgG titer was measured weekly through tail bleeding. Anti‐BPTI‐19A IgG levels for the C5P and C5R tagged BPTI‐19A sustained during the 3 weeks (Fig. 4C,D), and a booster dose (5th dose) of the respective variants injected 21 days after the 4th dose increased the IgG titers by 334‐ and 346‐fold, respectively (Table S2). On the other hand, the IgG titer of mice immunized with the untagged BPTI‐19A remained nil throughout the entire immunization period (Fig. 4C,D). Thus, in the absence of adjuvant, the immunogenicity increase generated by the non‐aggregating BPTI‐C5P and BPTI‐C5R was not as outstanding as that of the BPTI‐C5I [15] but nevertheless significant, and the higher titers were maintained for a couple of weeks (Fig. 4B–D).

### Short peptide tag: a biotechnological tool for increasing immunogenicity

This study was initiated in order to complement our previous ones, where we demonstrated that protein's immunogenicity could be boosted by attaching an SCP‐tag that oligomerizes the protein. Here, the immunogenicity improvement induced by the proline and the arginine tags probably involve mechanisms distinct from that generated by the C5I tag, which acts through the oligomerization of the antigens [14, 15, 16, 17]. However, from an application viewpoint, the critical finding is the noticeable increase of BPTIs' immune response generated by the C5P tag. On the other hand, 5 prolines attached at the N terminus (N5P) had virtually no effect on the immunogenicity of BPTI‐19A, and the C terminus 7‐proline (C7P) increased the immunogenicity to a lesser extent than expected from the C5P tag.

Thus, we hypothesize that the molecular mechanisms of the C5P‐induced immunogenicity enhancement might be different from the previously reported high immunogenicity of proline‐rich epitopes because the epitopes were located within the protein [37, 38] which was in contrast to our observation that the immune response increased only with the 5‐pro attached at the C terminus of the protein. We also hypothesize that the 5‐arg might have enhanced the entry of BPTI into the cells during the endocytosis/phagocytosis process [39, 40, 41], which might have increased the immunogenicity [42] of BPTI. Finally, the immunogenicity increase generated by the IRIRI and ININI was lower than C5R, C5N, or C5I, indicating that the effect of the type of amino acid (contained in the tags) on immunogenicity was not additive.

To the best of our knowledge, this is the first systematic analysis of the effects of short peptide tags on the immunogenicity of a small, non‐immunogenic protein. We found that the C5P and C5R tags could noticeably increase the immune response of BPTI‐19A without oligomerizing the protein. Importantly, we performed a “near‐real‐time” monitoring of the aggregates' state of the immunization sample by measuring the hydrodynamic radii just prior to each injection. This near‐real‐time monitoring was essential to reliably assess the absence of aggregates directly in the immunization samples, because aggregates may grow rapidly and randomly upon small variations of external conditions. Although further studies analyzing the mechanisms of the immunogenicity increase generated by the C5P and C5R tags are needed, our present observations suggest their strong potentials as a biotechnological tool for producing antibodies used in biopharmaceutical research.

## Conflict of interest

The authors declare no conflict of interest.

## Author contributions

YK, MMI, and NR designed the project. NR and MMI performed the experiments, analyzed, and compiled the data. NR wrote the manuscript with YK. MGK helped with the mutational analysis and manuscript preparation. SU performed and analyzed the AUC experiment. All authors read and approved the manuscript.

## Supporting information


**Fig. S1.** Analysis and comparison of biophysical properties of C5R, C5K, C5N, C5H, C5D, C5P, N5P, C7P, IRIRI, ININI and C5I tagged BPTIs with the untagged‐19A. (A) DLS spectra at 25 °C of the size distribution shown as number mean. (B) SLS spectra of BPTI variants at 25°C. (C) CD spectra of all BPTI variants at 25°C. (D) Tyr‐fluorescence and (E) ANS‐fluorescence spectra of untagged‐19A, C5R, C5K, C5N, C5H, C5D, C5P, N5I, C7P, IRIRI, ININI and C5I tagged variants at 25°C. (F) ThT‐fluorescence intensity of the tagged variants measured at 37°C where lysozyme was used as a positive control. SCP‐tagged variants were formulated at 0.3 mg/mL concentrations in PBS, pH 7.4 for DLS, SLS, CD and fluorescence measurements. Values are shown as the average of three independent measurements (DLS) and three accumulations (SLS, CD and fluorescence), respectively. Line symbols are explained within the panels. The error bars represent the standard deviation (SD).
**Fig. S2.** Dose‐dependent titer values of all mice injected with C5R, C5K, C5N, C5H, C5D, C5P, N5P, C7P, IRIRI, ININI and C5I tagged BPTIs and untagged BPTI‐19A (A) in the presence (+) and (B) absence (‐) of adjuvant. The error bars represent the standard deviation (SD).
**Fig. S3.** Dose‐dependent OD values of anti‐BPTI sera and comparison of the antibody titers when injections were carried out with and without adjuvant. (A) Dose‐dependent (with adjuvant) OD values of C5R, C5K, C5N, C5H, C5D, C5P, IRIRI and ININI tagged variants and the untagged BPTI‐19A at 492 nm determined by ELISA using the 4th tail‐bleeding serum samples. Values are the average of duplicated samples. Line symbols are explained within the panels. (B) Comparison of antibody titers of C5P, C5R, and C5I tagged BPTIs and the untagged‐19A injected with or without adjuvants. Outliers (open circles) were removed when computing the average IgG titer (grey bars), which was calculated using data from the high responsive mice (closed circles). (C) OD values of sera (using adjuvants) raised against untagged‐19A, C5R and C5P tagged BPTIs against the coating antigens of untagged BPTI‐19A and their respective tag, self‐tags. The error bars represent the standard deviation (SD).
**Fig. S4.** A schematic representation of the purification of SCP‐tagged BPTI proteins (RT‐room temperature (25°C); O/N‐overnight).
**Fig. S5.** Analysis of the oligomeric state of the BPTI proteins by analytical ultracentrifugation (AUC). AUC data analysis was performed using the continuous distribution c(s) analysis module in the SEDFIT program. Distribution of sedimentation coefficient c(s) for the BPTI proteins were obtained, then the “s” values were corrected to standard conditions (water and 20 degree), s_20,w_. In all of the panels, except for C5I, there was only one large, sharp peak in the c(s_20,w_) distribution, corresponding to the BPTI variant. This indicates that all SCP‐tagged BPTI variants were in a very pure monomeric state. For BPTI‐C5I, which was used as a positive control previously reported to form subvisible oligomers, a larger sedimentation coefficient than the monomer was observed.
**Table S1.** Hydrodynamic radius of BPTI‐19A and its SCP‐tagged variants. DLS measurements were carried out just before immunization at 25°C followed by 37°C at 0.3 mg/mL concentrations in PBS. The values are averaged over three independent measurements, and the errors are standard deviation (SD). *R*_h_ was calculated from the number‐distributions using the Stokes‐Einstein equation.
**Table S2.** Maintenance of IgG titers against BPTI‐19A, BPTI‐C5R, and BPTI‐C5P in the absence of adjuvant. ^1^The titers were calculated using a power fitting model, and the values were averaged using the number of the mice (n) in the respective groups [19A (‐), n=3 and C5R (‐), n=4, and C5P (‐), n=4]. ^2^Fold‐increase with respect to the titer of BPTI‐19A.
**Table S3.** Limulus amebocyte lysate (LAL) endotoxin assay of SCP‐tagged BPTI variants.

## Data Availability

All data generated or analyzed during this study are included in the article and its Supporting Information file. The structure of SCP‐tagged BPTI was generated using modeller (PDB ID: 3AUB).
