# Supplementary material for: A systematic mutational analysis identifies a 5‐residue proline tag that enhances the in vivo immunogenicity of a non‐immunogenic model protein
Source: FEBS Open Bio. 2020 Aug 30;10(10):1947–56. doi: 10.1002/2211-5463.12941 (PMC7530378; doi:10.1002/2211-5463.12941)
Supplement: Supplementary file 1 — Fig. S1. Analysis and comparison of biophysical properties of C5R, C5K, C5N, C5H, C5D, C5P, N5P, C7P, IRIRI, ININI and C5I tagged BPTIs with the untagged‐19A. (A) DLS spectra at 25 °C of the size distribution shown as number mean. (B) SLS spectra of BPTI variants at 25°C. (C) CD spectra of all BPTI variants at 25°C. (D) Tyr‐fluorescence and (E) ANS‐fluorescence spectra of untagged‐19A, C5R, C5K, C5N, C5H, C5D, C5P, N5I, C7P, IRIRI, ININI and C5I tagged variants at 25°C. (F) ThT‐fluorescence intensity of the tagged variants measured at 37°C where lysozyme was used as a positive control. SCP‐tagged variants were formulated at 0.3 mg/mL concentrations in PBS, pH 7.4 for DLS, SLS, CD and fluorescence measurements. Values are shown as the average of three independent measurements (DLS) and three accumulations (SLS, CD and fluorescence), respectively. Line symbols are explained within the panels. The error bars represent the standard deviation (SD). Fig. S2. Dose‐dependent titer values of all mice injected with C5R, C5K, C5N, C5H, C5D, C5P, N5P, C7P, IRIRI, ININI and C5I tagged BPTIs and untagged BPTI‐19A (A) in the presence (+) and (B) absence (‐) of adjuvant. The error bars represent the standard deviation (SD). Fig. S3. Dose‐dependent OD values of anti‐BPTI sera and comparison of the antibody titers when injections were carried out with and without adjuvant. (A) Dose‐dependent (with adjuvant) OD values of C5R, C5K, C5N, C5H, C5D, C5P, IRIRI and ININI tagged variants and the untagged BPTI‐19A at 492 nm determined by ELISA using the 4th tail‐bleeding serum samples. Values are the average of duplicated samples. Line symbols are explained within the panels. (B) Comparison of antibody titers of C5P, C5R, and C5I tagged BPTIs and the untagged‐19A injected with or without adjuvants. Outliers (open circles) were removed when computing the average IgG titer (grey bars), which was calculated using data from the high responsive mice (closed circles). (C) OD values of sera (using [file FEB4-10-1947-s001.docx]

**Supporting Information**

**A systematic mutational analysis identifies a 5-residue proline tag that enhances the *in vivo* immunogenicity of a non-immunogenic model protein**

Nafsoon Rahman^1*^, Mohammad Monirul Islam^2*^, Md. Golam Kibria^1^, Satoru Unzai^3^, Yutaka Kuroda^1#^

^1^Department of Biotechnology and Life Sciences, Graduate School of Engineering, Tokyo University of Agriculture and Technology, 2-24-16 Nakamachi, Koganei-shi, Tokyo 184-8588, Japan. ^2^Department of Biochemistry and Molecular Biology, University of Chittagong, Chittagong-4331, Bangladesh, ^3^ Department of Frontier Bioscience, Faculty of Bioscience and Applied Chemistry, Hosei University, 3-7-2 Kajino-cho, Koganei, Tokyo 184-8584, Japan.

**Supplementary Information**

**Figure S1:** Analysis and comparison of biophysical properties of C5R, C5K, C5N, C5H, C5D, C5P, N5P, C7P, IRIRI, ININI and C5I tagged BPTIs with the untagged-19A. (A) DLS spectra at 25°C of the size distribution shown as number mean. (B) SLS spectra of BPTI variants at 25°C. (C) CD spectra of all BPTI variants at 25°C. (D) Tyr-fluorescence and (E) ANS-fluorescence spectra of untagged-19A, C5R, C5K, C5N, C5H, C5D, C5P, N5I, C7P, IRIRI, ININI and C5I tagged variants at 25°C. (F) ThT-fluorescence intensity of the tagged variants measured at 37°C where lysozyme was used as a positive control for amyloid aggregates. SCP-tagged variants were formulated at 0.3 mg/mL concentrations in PBS, pH 7.4 for DLS, SLS, CD and fluorescence measurements. Values are shown as the average of three independent measurements (DLS) and three accumulations (SLS, CD and fluorescence), respectively. Line symbols are explained within the panels. The error bars represent the standard deviation (SD).

**Figure S2:** Dose-dependent titer values of all mice injected with C5R, C5K, C5N, C5H, C5D, C5P, N5P, C7P, IRIRI, ININI and C5I tagged BPTIs and the untagged BPTI-19A (A) in the presence (+) and (B) absence (-) of adjuvant. The error bars represent the standard deviation (SD).

**Figure S3:** Dose-dependent OD values of anti-BPTI sera and comparison of the antibody titers when injections were carried out with and without adjuvant. (A) Dose-dependent (with adjuvant) OD values of C5R, C5K, C5N, C5H, C5D, C5P, IRIRI and ININI tagged variants and the untagged BPTI-19A at 492 nm determined by ELISA using the 4^th^ tail-bleeding serum samples. Values are the average of duplicated samples. Line symbols are explained within the panels. (B) Comparison of antibody titers of C5P, C5R, and C5I tagged BPTIs and the untagged-19A injected with or without adjuvants. Outliers (open circles) were removed when computing the average IgG titer (grey bars), which was calculated using data from the high responsive mice (closed circles). (C) OD values of sera (using adjuvants) raised against untagged-19A, C5R and C5P tagged BPTIs against the coating antigens of untagged BPTI-19A and their respective tag, self-tags. The error bars represent the standard deviation (SD).

**Figure S4:** A schematic representation of the purification of SCP-tagged BPTI proteins (RT-room temperature (25°C); O/N-overnight).

**Figure S5:** Analysis of the oligomeric state of the BPTI proteins by analytical ultracentrifugation (AUC). AUC data analysis was performed using the continuous distribution c(s) analysis module in the SEDFIT program. Distribution of sedimentation coefficient c(s) for the BPTI proteins were obtained, then the “s” values were corrected to standard conditions (water and 20 degree), s_20,w_. In all of the panels, except for C5I, there was only one large, sharp peak in the c(s_20,w_) distribution, corresponding to the BPTI variant. This indicates that all SCP-tagged BPTI variants were in a very pure monomeric state. For BPTI-C5I, which was used as a positive control previously reported to form subvisible oligomers, a larger sedimentation coefficient than the monomer was observed.

**Table S1: Hydrodynamic radius of BPTI-19A and its SCP-tagged variants.** DLS measurements were carried out just before immunization at 25°C followed by 37°C at 0.3 mg/mL concentrations in PBS. The values are averaged over three independent measurements, and the errors bars represent standard deviation (SD). *R*_h_ was calculated from the number-distributions using the Stokes-Einstein equation.

**Table S2: Maintenance of IgG titers against BPTI-19A, BPTI-C5R, and BPTI-C5P in the absence of adjuvant.** ^1^ The titers were calculated using a power fitting model, and the values were averaged using the number of the mice (n) in the respective groups [19A (-), n=3 and C5R (-), n=4, and C5P (-), n=4]. ^2^Fold-increase with respect to the titer of BPTI-19A.

**Table S3: Limulus amebocyte lysate (LAL) endotoxin assay of SCP-tagged BPTI variants.**

**Table S1:** **Hydrodynamic radius of BPTI-19A and its SCP-tagged variants**

| **Mutant Identities** | **Average hydrodynamic radius (*R*_h,_ nm) in PBS** | |
| --- | --- | --- |
|  | 25°C | 37°C |
| BPTI-19A | 1.33±0.02 | 1.34±0.02 |
| BPTI-C5R | 1.54±0.03 | 1.44±0.05 |
| BPTI-C5K | 1.41±0.05 | 1.4±0.09 |
| BPTI-C5H | 1.39±0.06 | 1.38±0.14 |
| BPTI-C5D | 1.44±0.06 | 1.48±0.04 |
| BPTI-C5N | 1.38±0.09 | 1.4±0.09 |
| BPTI-C5P | 1.37±0.09 | 1.4±0.07 |
| BPTI-N5P | 1.28±0.08 | 1.5±0.08 |
| BPTI-C7P | 1.44±0.04 | 1.53±0.09 |
| BPTI-IRIRI | 1.23±0.09 | 1.41±0.15 |
| BPTI-ININI | 1.16±0.07 | 1.32±0.07 |
| BPTI-C5I | 3.2±0.06 | 3.71±0.13 |

| **Mutants** | **Tags** | **Average titer after booster dose (D-52) ^1^** | **Fold increased^2^** |
| --- | --- | --- | --- |
| BPTI-19A | **×** | 5.92 | 1 |
| BPTI-C5R | Gly_2_ Arg_5_ | 2053.25 | 346.55 |
| BPTI-C5P | Gly_2_Pro_5_ | 1981.96 | 334.52 |

**Table S2: Maintenance of IgG titers against BPTI-19A, BPTI-C5R, and BPTI-C5P in the absence of adjuvant**

| **Potent SCP-tagged variants** | **EU/mL** | **EU/kg/h** |
| --- | --- | --- |
| BPTI-C5R | 0.17 | 1.36 |
| BPTI-C5P | 0.59 | 4.73 |
| BPTI-C7P | 0.18 | 1.46 |
| BPTI-C5I | 0.47 | 3.75 |

**Table S3: Limulus amebocyte lysate (LAL) endotoxin assay of SCP-tagged BPTI variants**

**
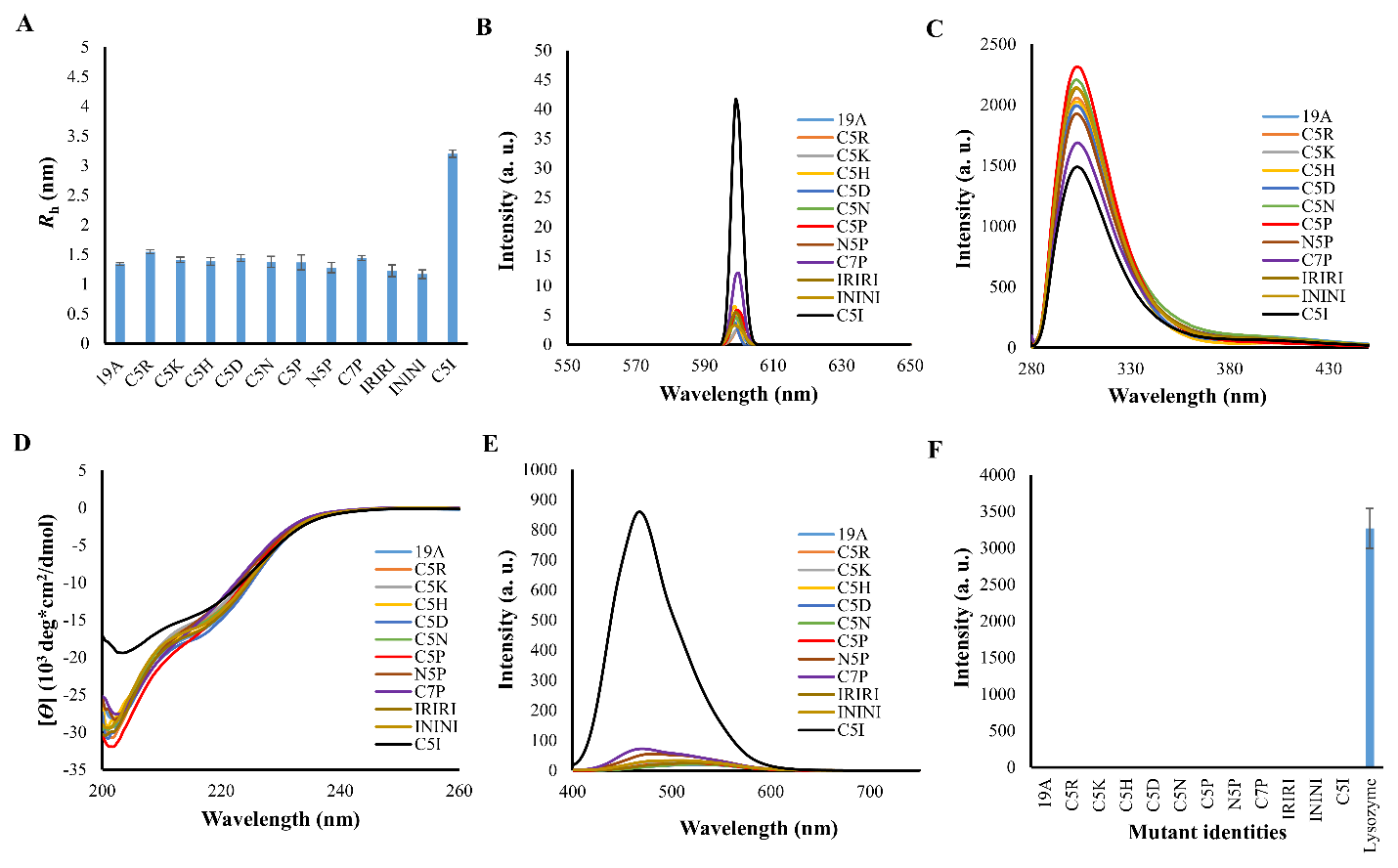
Figure S1**

**Figure S2**

**
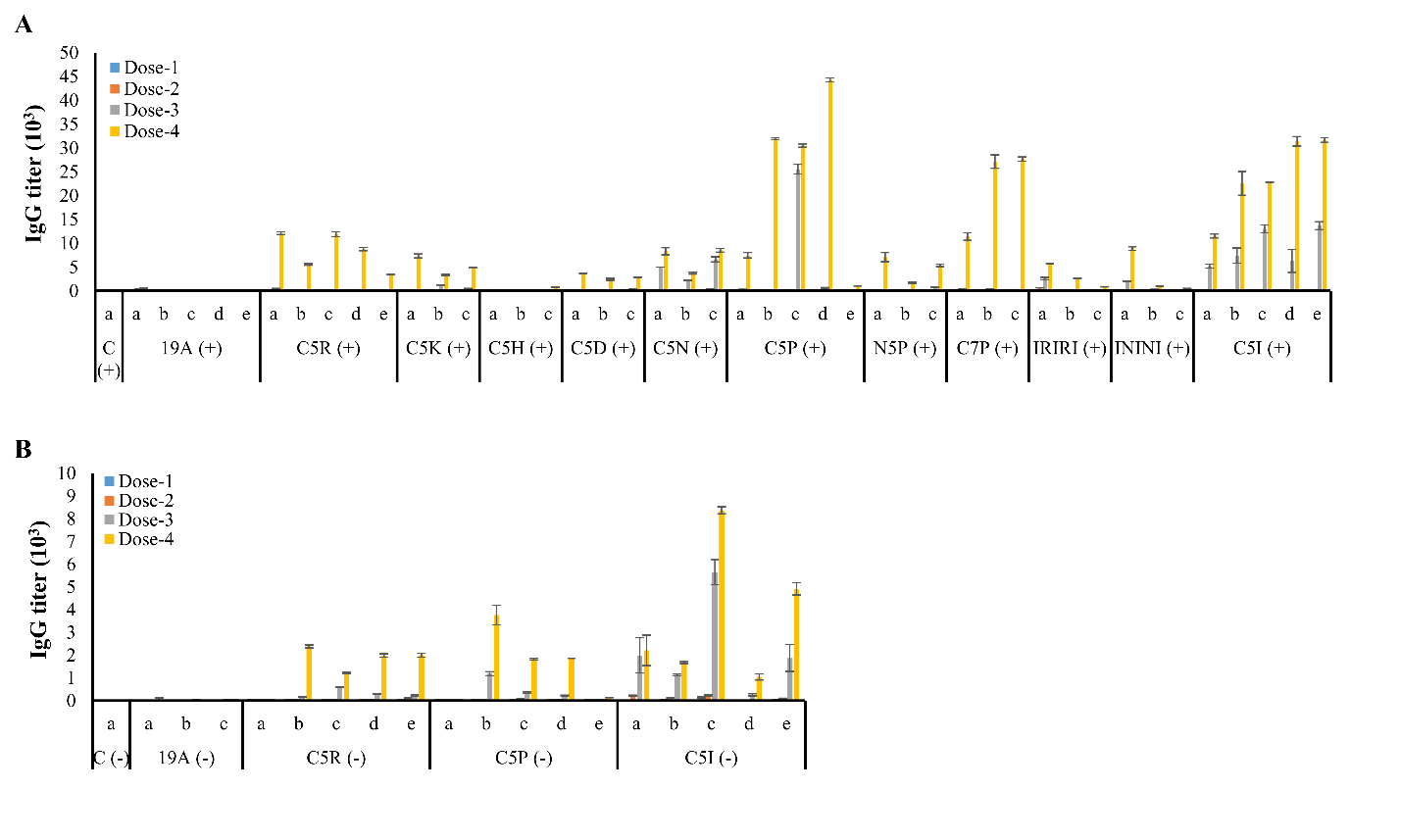
**

**Figure S3**


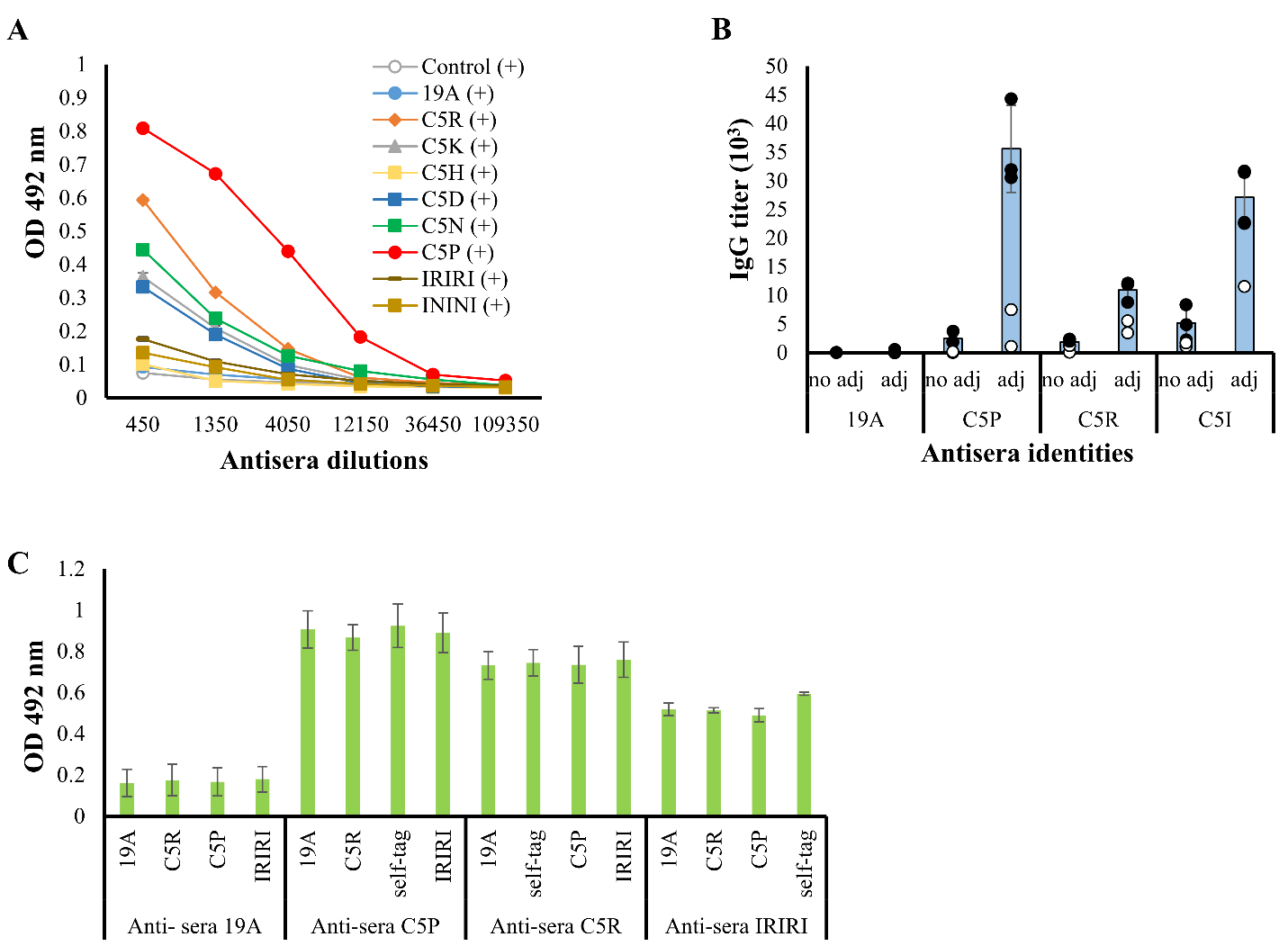


**Figure S4**


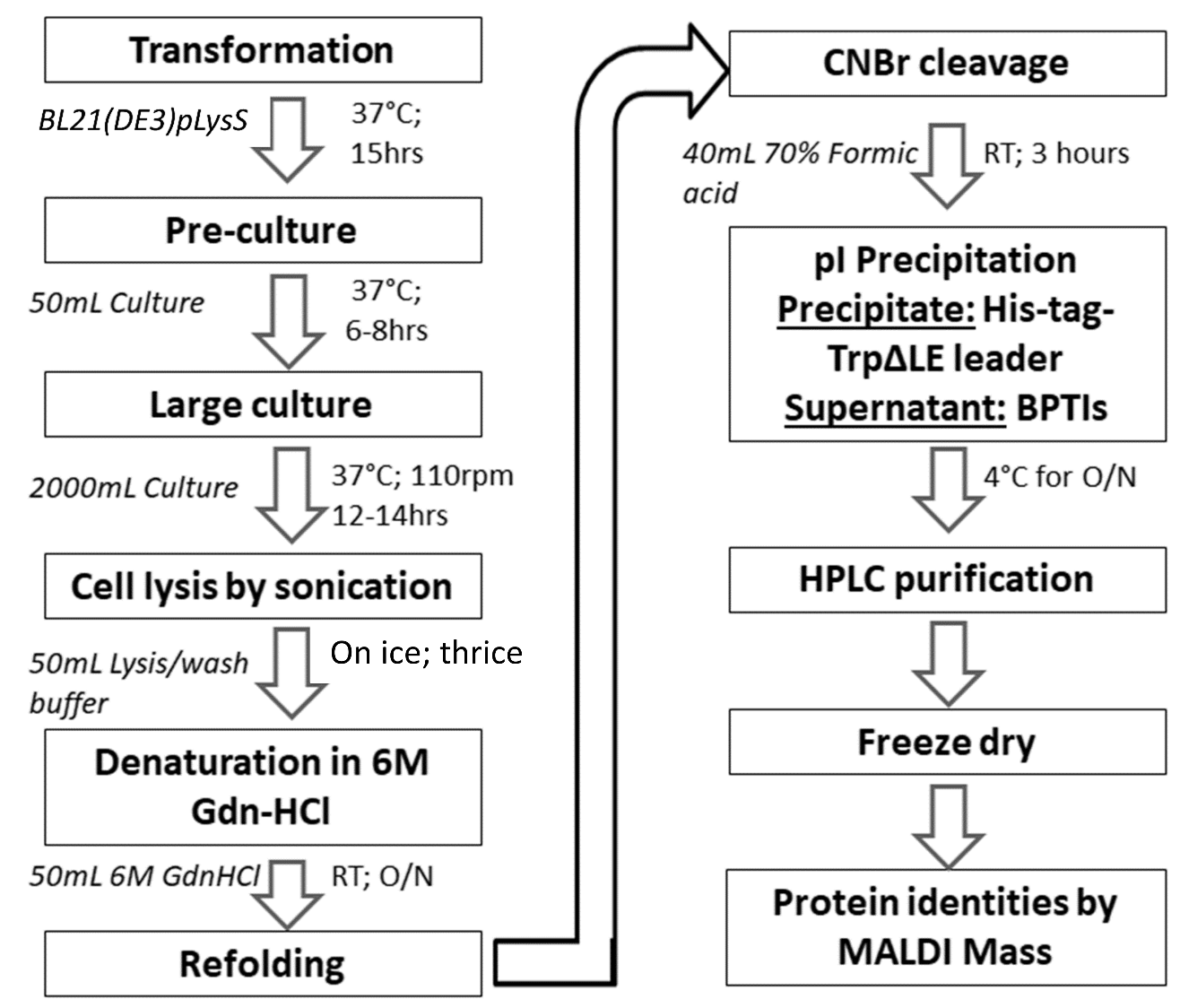


**Figure S5**

**
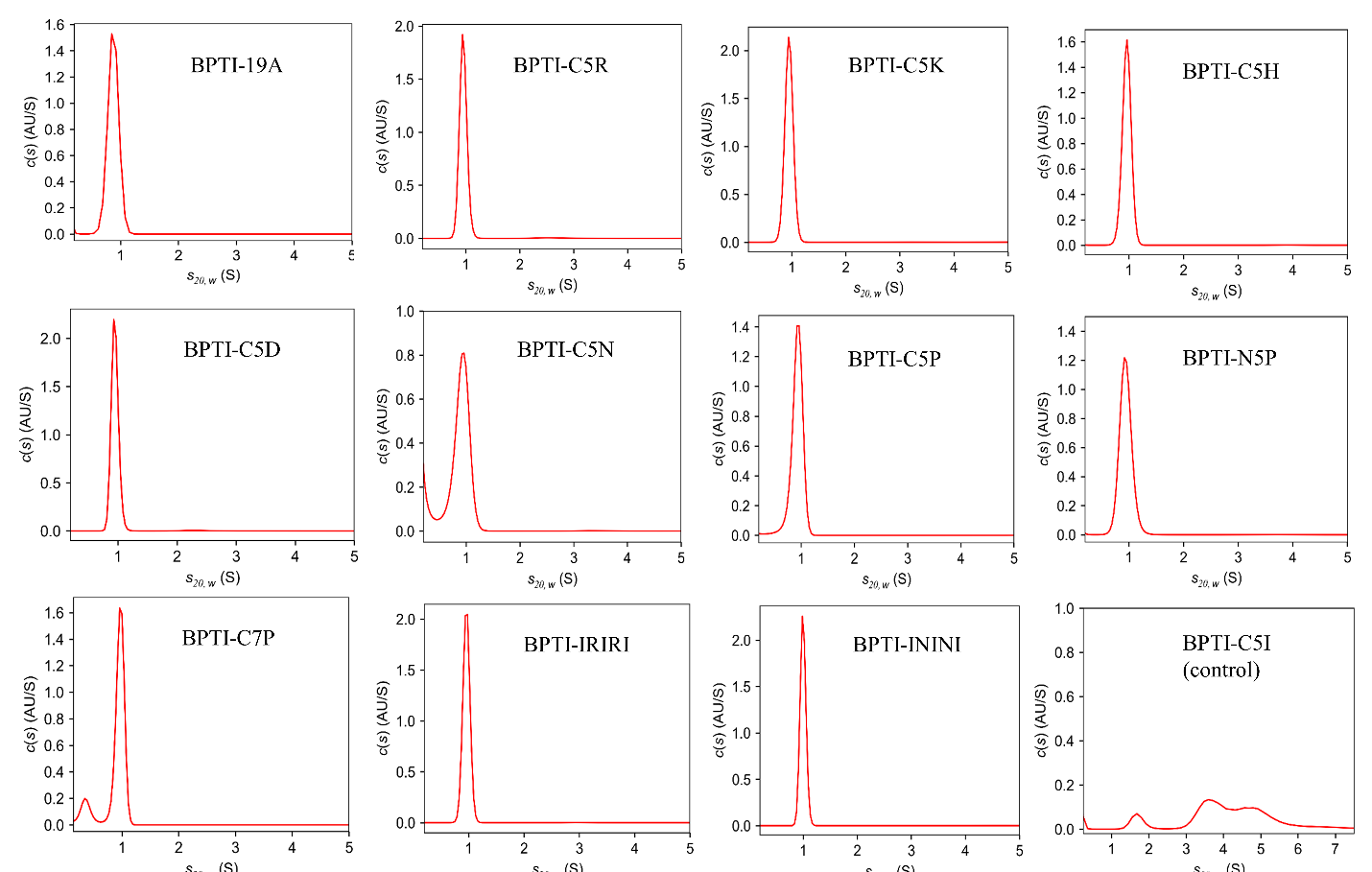
**
